# A Case Report of a Perforated De Garengeot Hernia

**DOI:** 10.7759/cureus.98089

**Published:** 2025-11-29

**Authors:** Shawn Brain, Randa Barsoom

**Affiliations:** 1 College of Osteopathic Medicine, Touro College of Osteopathic Medicine, Middletown, USA; 2 Department of General Surgery, Montefiore Nyack Hospital, Nyack, USA

**Keywords:** acute abdomen, appendicitis, case report, de garengeot hernia, femoral hernia, general surgery, incarcerated hernia, mesh repair, robotic surgery, strangulated hernia

## Abstract

De Garengeot hernias are defined as the presence of an incarcerated appendix within a femoral hernia. Patients typically present with a painful and irreducible groin bulge, often without classic signs of appendicitis, and ambiguous imaging, making preoperative diagnosis difficult. Further, with no standardized management strategy, treatment is based on case presentation and surgeon preference. Here, we report a rare case of an intraoperatively diagnosed perforated De Garengeot hernia and how it was successfully managed.

A 55-year-old female patient presented with a three-week history of a progressively worsening, erythematous, severely tender right groin bulge with mild abdominal pain. She was febrile, and the physical exam demonstrated a nonreducible groin mass without any peritoneal signs. CT abdomen/pelvis and ultrasound of the right groin suggested an incarcerated femoral hernia likely composed of strangulated fat with overlying cellulitis. The patient was taken for robotic femoral hernia repair, during which a De Garengeot hernia was identified. The case continued with a robotic appendectomy with subsequent incision and removal of the appendix over the femoral canal. Due to significant contamination, hernia repair with mesh was deferred. A Penrose drain was placed, and the patient recovered uneventfully with plans for future repair.

De Garengeot hernias are exceptionally rare pathologies, often presenting with nonspecific symptoms and inconclusive imaging. Thus, diagnosis is difficult and often done intraoperatively. This case emphasizes the need to maintain a high clinical suspicion and an appropriate readiness to convert strategy if discovered.

## Introduction

A De Garengeot hernia is defined as the presence of an incarcerated vermiform appendix within a femoral hernia sac [1,2]. This is a rare pathology, with uncomplicated femoral hernias constituting approximately 3-4% of all groin hernias and De Garengeot hernias representing less than 1% of all femoral hernias [3]. When further complicated by appendicitis (seen in ~44.1-84.4% of cases) and perforation (seen in ~10% of cases) within the sac, as seen in this patient, the incidence becomes extraordinarily lower [1,4].

Typical presentation of De Garengeot hernias consists of an acutely tender, erythematous, nonreducible groin bulge, most often in elderly women. Systemic signs such as fever occur in approximately 39% of patients, and leukocytosis is present in approximately two-thirds of cases [1,2,4]. Despite presenting as two separate surgical emergencies in one (appendicitis and an incarcerated femoral hernia), they are rarely diagnosed prior to surgical intervention. Meta-analyses demonstrate that accurate preoperative identification occurs in only 14-31.5% of cases [1,2]. This is likely attributed to a low index of suspicion, nonspecific clinical presentation, and limitations of imaging modalities [5].

Furthermore, treatment of De Garengeot hernias has yet to become standardized, with a variety of approaches taken, depending on the specific case and surgeon preference [4]. This report describes a complicated case of a De Garengeot hernia, consisting of an intraoperative diagnosis, appendicitis, strangulation, and perforation. It also demonstrates the approach taken in such a situation, emphasizing infection control and patient safety.

## Case presentation

A 55-year-old female patient presented to the emergency department with a three-week history of a right groin bulge associated with abdominal pain. The patient reported visiting another hospital's emergency room at the onset of symptoms, where she had a CT abdomen/pelvis that demonstrated no acute appendicitis, only noting a fluid collection in the right groin and dilated venous collaterals in the medial right thigh. She was discharged with a preliminary diagnosis of a canal of Nuck cyst and instructions for an outpatient surgical follow-up. She reported that the symptoms continued to progress over the following weeks, developing into severe, unrelenting abdominal pain with progressive enlargement and erythematous changes of the groin bulge, prompting her subsequent emergency room visit. On arrival, the patient was febrile to 100.6°F and mildly hypertensive (blood pressure of 142/69 mmHg). All other vital signs were within normal limits. Physical examination revealed a nonreducible, erythematous, firm bulge in the right groin. The abdomen was tender to palpation but nondistended, with the patient reporting a normal bowel movement earlier that day.

**Figure 1:**
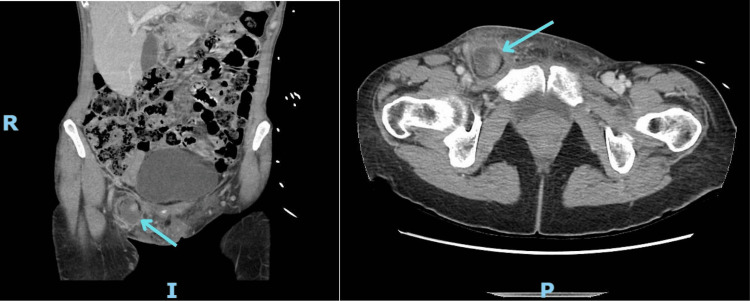
Coronal and axial cuts of the femoral hernia visualized on computed tomography (CT) scan of the patient

Laboratory evaluation demonstrated normocytic anemia and mild neutrophilia on complete blood count (CBC). The rest of the results were within normal limits, including basic metabolic panel (BMP), liver function tests (LFTs), coagulation studies, blood cultures, lactate, and procalcitonin. With suspicion of an incarcerated and possibly strangulated groin hernia, the patient was re-evaluated with a CT abdomen/pelvis with IV contrast. The radiology report demonstrated inflammatory changes in the right groin with a 2.9 cm hypodense structure in the right inguinal canal, possibly representing a fluid collection or vascular structure (Figure 1).

This prompted further evaluation with an ultrasound of the pelvis and one overlying the bulge on the right groin. This confirmed the presence of a femoral hernia and reported it as likely containing strangulated fat with overlying cellulitis versus reactive changes.

**Figure 2:**
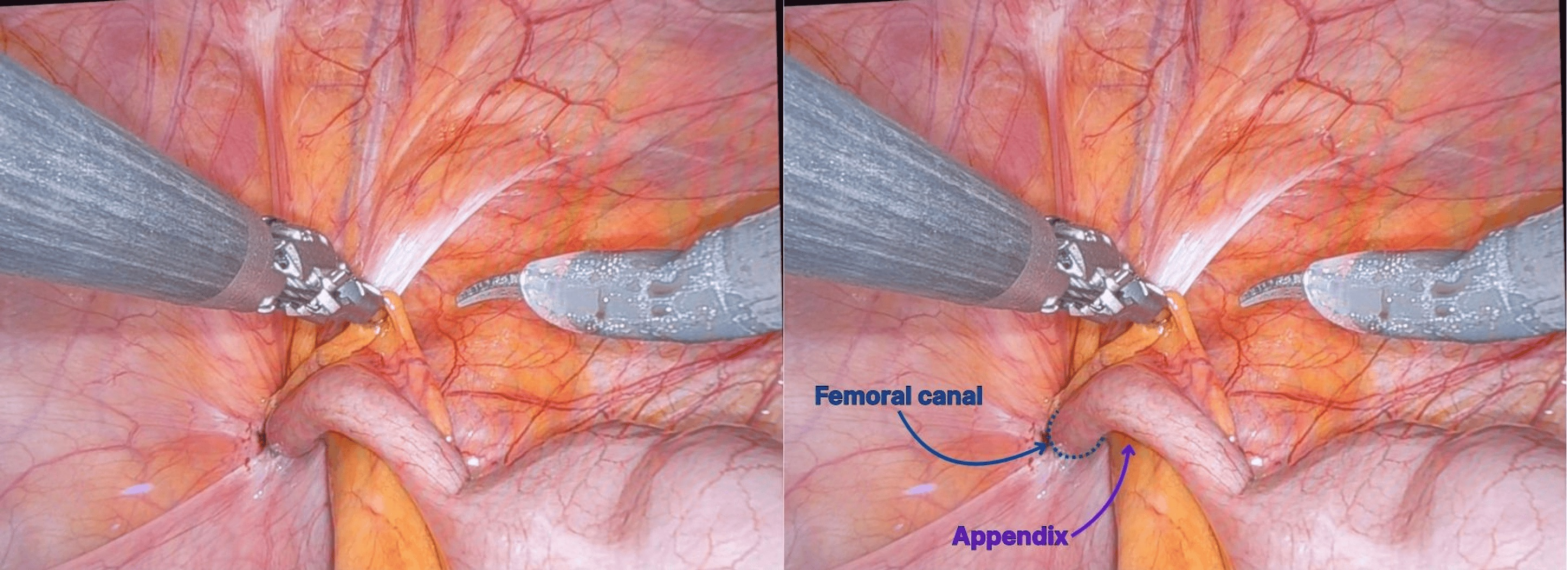
Intraoperative visualization of the appendix incarcerated within the femoral hernia (De Garengeot hernia) with robotic assistance

Written informed consent has been obtained from the patient, and she was taken to the operating room for robotic repair of a suspected incarcerated femoral hernia. After docking the robot and visualizing the peritoneal cavity, a De Garengeot hernia was identified, with the appendix clearly incarcerated in the femoral canal (Figure 2).

**Figure 3:**
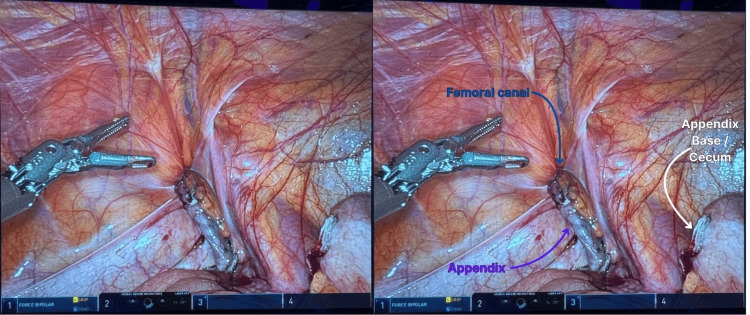
Robotic-assisted appendectomy showing incarcerated appendix within the femoral hernia

Attempts at robotic and manual reduction were unsuccessful due to dense adherence of the appendiceal tip. Therefore, the decision was made to proceed with a robotic appendectomy, with the appendix base localized and stapled off (Figure 3).

**Figure 4:**
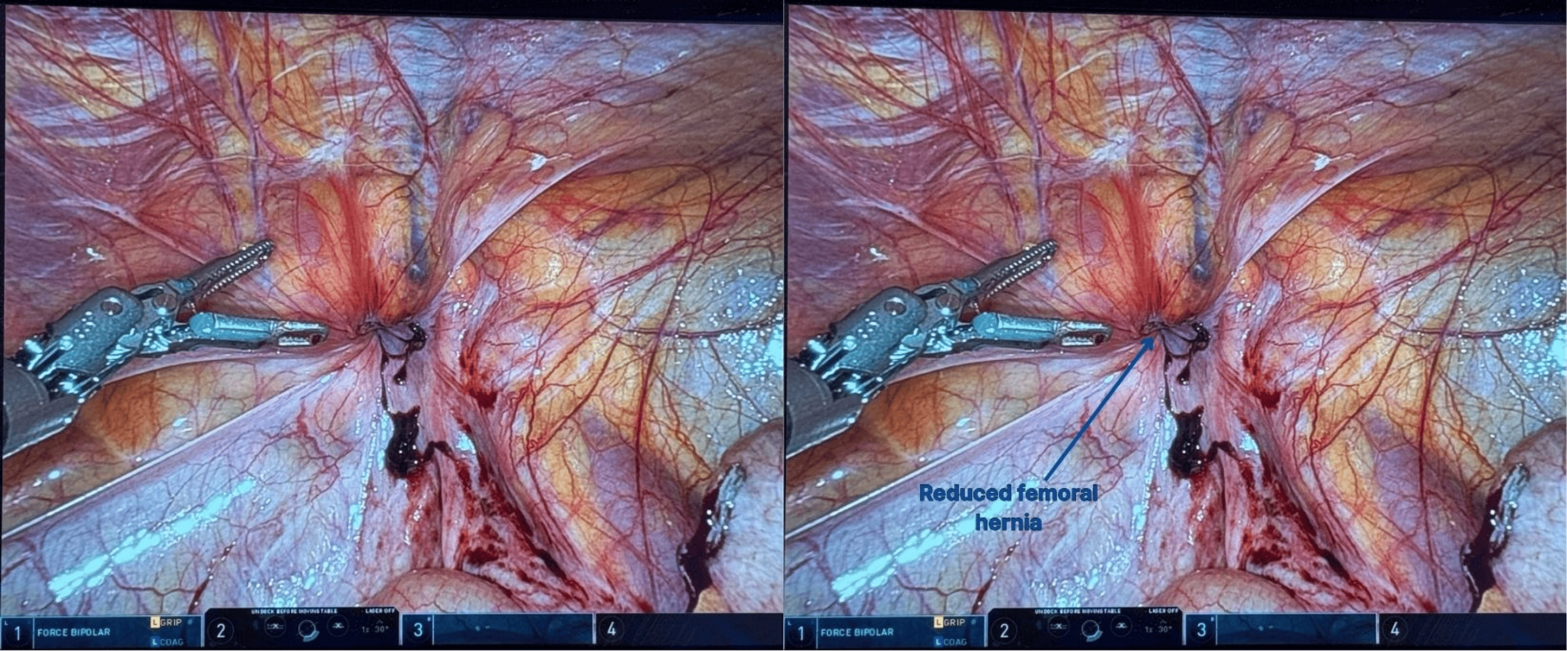
Residual hernia defect after reduction of the appendix through the external opening of the femoral canal

A second attempt at intraperitoneal reduction of the hernia was unsuccessful; thus, conversion to an external approach took place due to concern for perforation and abscess formation within the femoral canal. This approach allowed controlled drainage and cleaning of the contaminated space while minimizing the risk of peritoneal contamination. With the robot remaining docked, an incision was made directly over the groin bulge. A perforated appendix with purulent drainage was visualized, and the inflammation was suctioned off. The appendix was dissected from the surrounding structures, extracted through the external incision, and the hernia sac was ligated (Figure 4).

Due to purulent contamination, definitive hernia repair with mesh was deferred. A Penrose drain was placed below the skin for ongoing drainage, and the subcutaneous incision was closed. The surgeon returned to the robotic console to thoroughly irrigate and suction the contaminated peritoneal cavity before undocking the hardware and closing all incisions.

The patient was discharged on postop day 2 without any complications. Pathologic examination of the intraoperative specimen revealed a 7.5 cm appendix with necrosis and abscess formation confined to the appendiceal tip, along with separate fragments of purulent exudate. The patient made a complete recovery, and the Penrose drain was removed 10 days postop. She was seen once more two weeks after that, demonstrating adequate wound healing and reporting a near-complete return to activities of daily living. The decision was made for her to return for an elective femoral hernia repair with mesh within the following three months.

## Discussion

De Garengeot hernias are a rare pathology, estimated to make up around 1% of all femoral hernias, with only ~450 cases reported since their first identification in 1731 [1,6]. Furthermore, perforation of the appendix within the hernia sac is a complication seen in less than 10% of cases, adding a layer of complexity to our case [4,7]. The pathophysiology delineating the migration of the appendix into the femoral hernia is poorly understood. Notable hypotheses consist of abnormal cecal orientations, long appendices, and increased intra-abdominal pressure, with the real explanation likely being multifactorial [3]. Postpartum anatomical changes, such as femoral ring enlargement, are often attributed, explaining the increased prevalence seen in females (4:1 female-to-male ratio) [2]. Additionally, the inflammatory progression was likely secondary to the extraluminal constriction of the appendix by the hernial opening [8]. This inevitably leads to the development of complications, including strangulation, necrosis, perforation, and possible sepsis. 

Our patient presented with typical signs of a De Garengeot hernia, consisting of a tender (79.8% of cases), erythematous (33.3% of cases), and bulging groin mass (82.4% of cases) [1]. She also presented with late onset and nonspecific systemic symptoms, including a low-grade fever and diffuse abdominal pain. It was unclear if those were related to the inflammation, strangulation, and/or perforation of the appendix. Absence of peritonitis and/or a profound systemic response (i.e., leukocytosis, high fever, tachycardia, nausea, vomiting, etc.) was likely due to the containment of infection within the femoral sac [3]. This disproportionate and delayed presentation makes the diagnosis of De Garengeot hernias a challenge, contributing to their frequent diagnostic delay [2].

Although imaging can aid in preoperative recognition, it is shown to be unreliable, as further exhibited by our case [5]. A CT scan is considered to carry the highest efficacy when diagnosing De Garengeot hernias. However, it demonstrates a relatively poor 61.0% sensitivity when compared to the 95% sensitivity for the diagnosis of a typical appendicitis [1,9]. Our patient underwent two CT scans of the abdomen and pelvis and two ultrasounds, all of which failed to localize the appendix within the femoral hernia. Upon retrospective review, the hypodense structure seen on imaging was confirmed to contain a high-attenuation tubular structure, which corresponds to the appendix. However, its continuity with the intra-abdominal bowel was not appreciated prospectively due to surrounding inflammation and limited image slices at the level of the hernia, obscuring the anatomy. This is often the situation for De Garengeot hernias, with less than one-third of cases being identified preoperatively [1,2,10]. Overall, awareness of this condition is crucial for surgeons evaluating patients with nonreducible groin masses as well as radiologists reviewing the imaging in these cases. Maintaining a level of suspicion for complicated hernias, such as De Garengeot hernias, can prevent diagnostic delays and guide preoperative planning.

Although definitive management principles do not exist, treatment consists of emergent appendectomy and hernia repair, with the sequence and technique dependent on intraoperative findings, the degree of contamination, and surgeon preference [1,4,8]. If the appendix is unperforated, simultaneous appendectomy and hernia repair with mesh may be performed safely. In contrast, perforated cases pose a high risk for mesh infection; thus, primary tissue repair or delayed mesh placement is preferred [2,11]. Our case exhibited a class 5 De Garengeot hernia (perforated appendix, abscess, or fistula) displaying a contaminated site [1,12]. This prompted deferral of the hernia repair, which is consistent with recommended surgical judgment to minimize postoperative complications such as mesh infections, surgical site infections, wound dehiscence, recurrence of hernias, and sepsis [11].

This case also highlights the growing role of minimally invasive and robotic techniques in managing complex groin hernias. The robotic platform provided excellent visualization of the femoral canal for diagnosis and allowed for safe dissection, stapling, and irrigation while maintaining precision. Although open approaches remain standard in emergent settings, literature increasingly supports laparoscopic and robotic methods for both diagnosis and treatment of atypical hernias [1,3,13]. The primary limitation of this case report includes the incomplete course of treatment, with femoral hernia repair with mesh pending in the following months.

## Conclusions

De Garengeot hernias with perforated appendicitis are exceptionally rare surgical entities that pose both diagnostic and therapeutic challenges. This case highlights the utility of robotic surgery for accurate visualization and safe management, and the importance of individualized operative strategy, prioritizing infection control and staged repair to address both pathologies effectively. Physicians should maintain a high index of suspicion for this diagnosis in patients presenting with incarcerated femoral hernias, as timely recognition and appropriate surgical planning are key to favorable outcomes.
